# High‐intensity interval training in breast cancer patients: A systematic review and meta‐analysis

**DOI:** 10.1002/cam4.6387

**Published:** 2023-08-17

**Authors:** Xudong Chen, Xuyuan Shi, Zhiruo Yu, Xuelei Ma

**Affiliations:** ^1^ Department of Biotherapy, Cancer Center and State Key Laboratory of Biotherapy, West China Hospital Sichuan University Chengdu China; ^2^ West China School of Medicine, West China Hospital Sichuan University Chengdu China

**Keywords:** breast cancer, meta‐analysis, quality of life, women's cancer

## Abstract

**Background:**

Women with breast cancer and improved survival often experience treatment‐related impairments. High‐intensity interval training (HIIT) has emerged as a promising exercise therapy modality for adult cancer patients. However, the overall effects of HIIT in breast cancer patients remain scarce and controversial. Therefore, we conducted a systematic review and meta‐analysis to comprehensively evaluate the impact of HIIT on health‐related outcomes in breast cancer patients.

**Methods:**

We searched the PubMed, Embase, and Web of Science from inception to November 7, 2022. Eligible studies included randomized controlled trials that compared HIIT interventions with usual care (UC) or MICT in breast cancer patients. The primary outcome assessed was physical fitness, and exploratory outcomes included body composition, blood‐borne biomarkers, and patient‐reported outcomes. Summary data were extracted, and standardized mean differences (SMD) were calculated for meta‐analysis. For outcomes that could not be pooled, a systematic review was conducted.

**Results:**

Our analysis included 19 articles from 10 studies, encompassing 532 participants who met the inclusion criteria. Pooled results demonstrated that HIIT was superior to UC in improving peak oxygen uptake (VO_2peak_). The SMD for VO_2peak_ (L/min) and VO_2peak_ (mL/kg/min) was 0.79 (95% CI 0.13, 1.45) and 0.59 (95% CI 0.01, 1.16), respectively. No significant differences in VO_2peak_ were found between the HIIT and MICT groups. Meta‐analyses on body composition and blood‐borne biomarkers showed no significant differences between HIIT and UC. Systematic review indicated favorable effects of HIIT on muscle strength, fatigue, and emotional well‐being.

**Conclusions:**

HIIT is a time‐efficient alternative to MICT for improving VO_2peak_ and may also enhance muscle strength and alleviate fatigue and emotional symptoms in breast cancer patients. HIIT should be considered as an important component of exercise prescription in breast cancer care. Further studies with larger cohorts are needed to determine the clinical significance of HIIT‐induced changes in terms of other outcomes in women with breast cancer.

## 1 INTRODUCTION

Breast cancer has become the most prevalent cancer type worldwide, and is the leading cause of cancer‐related deaths in women.^1^ Nevertheless, the 5‐year survival rates of female breast cancer patients were 80% or higher and continued to increase in most countries.^2^ Despite these survival gains, a wide range of complications arises as a consequence of treatment interventions and changes in lifestyle activities. These complications have the potential to negatively impact prognosis, health‐related quality of life (HRQoL), as well as physical and psychosocial functions.^3^, ^4^, ^5^ Therefore, addressing posttreatment rehabilitation approaches becomes critical for the management of breast cancer and mitigating treatment‐related impairments.^6^

Exercise has demonstrated significant efficacy in improving overall survival rates following a breast cancer diagnosis.^7^, ^8^ Moreover, physical activity has been shown to be a safe and effective strategy for improving HRQoL, as well as psychological, behavioral, and physical outcomes in breast cancer patients undergoing and after adjuvant therapy.^9^, ^10^, ^11^ Patients are strongly encouraged to resume normal daily activities promptly after diagnosis and maintain regular engagement in physical activity, according to the ACS/ASCO 2016 Breast Cancer Survivorship Care Guideline. Specifically, breast cancer patients should aim for at least 150 min of moderate‐intensity exercise or 75 min of vigorous‐intensity exercise per week. Additionally, incorporating strength training exercises at least twice a week is recommended.^12^ However, there remains a lack of clarity regarding specific details such as exercise mode, frequency, intensity, and duration. Recent studies have highlighted various effective physical training methods in the rehabilitation treatment across different cancer types.^13^, ^14^, ^15^ Notably, high‐intensity interval training (HIIT) has demonstrated its cost‐effectiveness in adult cancer patients by reducing exercise time and significantly improving physical fitness and health outcomes.^16^ HIIT involves repeated intervals of high‐intensity effort, with an intensity of ≥90% of maximal oxygen consumption (VO_2max_) for healthy individuals or ≥80% VO_2max_ for clinical populations, followed by periods of low‐intensity or passive recovery.^17^, ^18^ This exercise modality has shown to elicit favorable physiological adaptations, including notable enhancements in VO_2peak_ within a short exercise duration when compared to UC and MICT.^19^, ^20^

Systematic reviews investigating the impact of HIIT in cancer patients, including a significant number of breast cancer cases, have consistently reported improved cardiorespiratory fitness and HRQoL.^21^, ^22^, ^23^ However, to date, no review has exclusively examined the effects of HIIT in patients with breast cancer. Therefore, in this study, we aim to integrate and analyze all existing articles on HIIT exercise interventions in breast cancer patients. The outcomes of interest included physical fitness, body compositions, blood‐borne biomarkers, and patient‐reported outcomes. Our objective is to comprehensively understand the overall effectiveness of HIIT in this specific population and provide valuable insights into exercise rehabilitation strategies for breast cancer patients.

## 2 METHODS

### 2.1 Search strategy and selection criteria

This study adhered to the Cochrane Collaboration guidelines for systematic reviews of interventions and followed the Preferred Reporting Items for Systematic Reviews and Meta‐Analyses (PRISMA) guidelines.^24^ Ethical approval or consent was not required as all data used were anonymous.

We conducted a comprehensive search of PubMed, Embase, and Web of Science from inception to November 2022, using three main domains: (1) high‐intensity interval training; (2) breast cancer patients; and (3) randomized controlled trial. The complete list of synonyms and the detailed search strategy are available in Table SA1. We also reviewed the reference lists of relevant systematic reviews to identify potential eligible studies that were not found in our regular database search.

Full‐text articles in the English language were included if they met the following criteria based on the PICOS principle: (1) participants were breast cancer patients or breast cancer survivors; (2) the intervention included HIIT with a clear description, and high intensities were defined a priori as ≥75% of VO_2peak_, peak heart rate (HRpeak), maximal heart rate (HRmax), or equivalent rating of perceived exertion (RPE) ≥16 on the BORG's 6–20 scale^25^; (3) the control group received UC or MICT^26^; (4) articles reported the outcomes of interest at both pre‐ and post‐interventional time; and (5) the study design was a randomized controlled trial. Additionally, when multiple articles were derived from the same clinical trial, we included the articles reporting specific parameters for the first time.

Two investigators (XC and XS) independently reviewed the search results. Irrelevant studies were excluded by reviewing the title and abstract according to the pre‐specified selection criteria, and potential articles were selected for full‐text screening. Any disagreements on eligibility were resolved through discussion between the two investigators. One study including participants with breast cancer and other categories of cancers was excluded after our discussion, because the result would be influenced by other cancers.

### 2.2 Study quality assessment

The quality of included articles was evaluated by two reviewers (XC and XS) separately, utilizing the Revised Cochrane risk‐of‐bias tool for randomized trials (RoB 2),^27^ which includes five domains for assessing the risk of bias. The algorithm was used to estimate the overall risk of bias of each article, which was categorized as high, some concerns, or low. Any discrepancies in the judgment of risk of bias were resolved through discussion among the two reviewers.

### 2.3 Data extraction and statistical analysis

Basic information from the included studies was extracted into a pre‐piloted Microsoft Excel sheet developed based on the PICOS principle by XC and XS after full‐text reading. The primary outcome was VO_2peak_, and the secondary outcome was other cardiopulmonary fitness indices, muscle strength, body composition, blood‐borne biomarkers, and patient reported outcomes. The two investigators independently completed the data extraction and checked with each other to reach a consensus.

We conducted meta‐analyses on outcomes that were reported in two or more studies, and for outcomes that were reported in only one study, they will be described in the result part. The meta‐analyses were performed using STATA 16.0, and we calculated the pooled estimate of SMD as all outcomes were continuous variables. We used Cohen's d to calculate the effect sizes of each endpoint, and we employed a random‐effects model. We evaluated the heterogeneity using the Chi‐squared test and *I* ^2^ statistics, and heterogeneity was considered significant if the *p*‐value was less than 0.10 or if *I* ^2^ was greater than 50%. We presented all combined results as SMD with its corresponding 95% confidence interval (95% CI) in the table. An SMD that overlapped with zero indicated that there was no significant difference between the different groups. We utilized Review Manager 5.4 software to generate the forest plot. For results that were only available in figure form in the original articles, we extracted the relevant data using Engauge Digitizer software.

## 3 RESULTS

### 3.1 Study selection and characteristics

**FIGURE 1:**
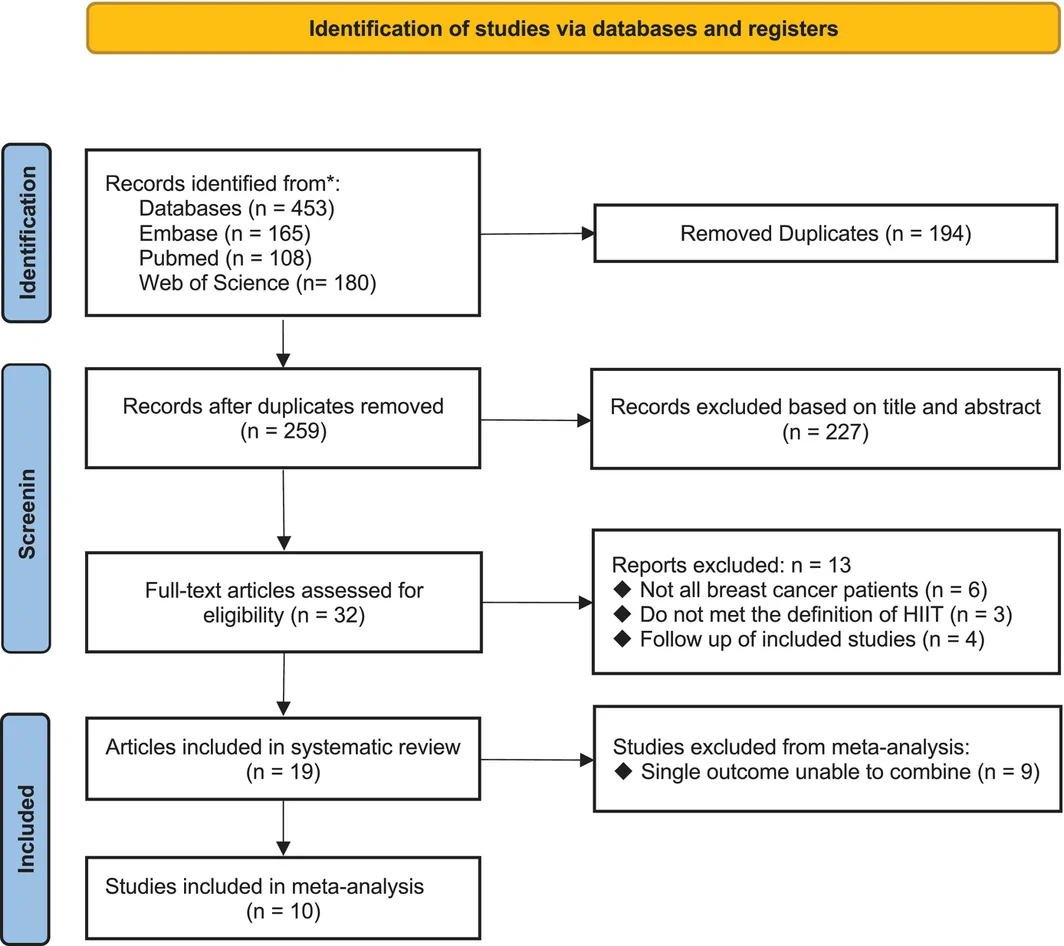
PRISMA flowchart of the systematic review process. PRISMA, Preferred Reporting Items for Systematic Reviews and Meta‐Analyses. *Consider, if feasible to do so, reporting the number of records identified from each database or register searched (rather than the total number across all databases/registers). **If automation tools were used, indicate how many records were excluded by a human and how many were excluded by automation tools.

A total of 429 articles were initially identified from three databases in our systematic literature search, as shown in Figure 1. Briefly, after removal of duplicates, 243 records were screened based on title and abstract, and 32 potential eligible articles were further examined in full‐text. Ultimately, 19 articles were included in the systematic review.^28^, ^29^, ^30^, ^31^, ^32^, ^33^, ^34^, ^35^, ^36^, ^37^, ^38^, ^39^, ^40^, ^41^, ^42^, ^43^, ^44^, ^45^, ^46^ In order to avoid duplication, for articles using the same study population from a single clinical trial, we only extracted information and data that appeared for the first time. Consequently, there were overall 10 usable studies for meta‐analysis.^32^, ^33^, ^35^, ^36^, ^39^, ^42^, ^43^, ^44^, ^45^, ^46^ The flow diagram outlines the specific reasons for exclusion. A summary of main characteristics of included articles is provided in Table 1. Details concerning exercise strategies and adherence are available in Table SA2.

**TABLE 1:** Basic characteristics of the included studies.

Study	Participants	Sample size (dropout)	Age (year) Mean, (SD)	Grouping	Outcomes	Timing	Design
Dolan, 2016	BCS	36 (3)	57.2 (9)	HIIT MICT UC	VO2peak, Body composition, Blood‐borne biomarkers	6 weeks	Pilot RCT
Mijwel, 2017	BCP planned to undergo chemotherapy	161 (29)	53.3 (10.3)	AT‐HIIT RT‐HIIT UC	Endurance and strength performance, QoL	16 weeks	RCT
Schulz, 2017	BCP	26 (0)	54 (8.9)	HIIT UC	VO_2peak_, Muscle strength, QoL, Training monitoring	6 weeks	pilot RCT
Mijwel, 2018a	BCP planned to undergo chemotherapy	40 (24)	51.2 (10.9)	AT‐HIIT RT‐HIIT UC	Skeletal muscle biopsies results	16 weeks	RCT
Northey, 2018	BCS	17 (0)	62.9 (7.8)	HIIT MICT UC	Cognitive performance, Resting cerebrovascular function, Resting blood pressure, Resting respiratory variables, VO_2peak_	12 weeks	pilot RCT
Mijwel, 2018b	BCP undergoing chemptherapy	240 (34)	57.7 (9.3)	AT‐HIIT RT‐HIIT UC	Cardiopulmonary fitness, Body composition, Muscle strength, Pressure‐pain threshold, Hemoglobin	16 weeks	RCT
Alizadeh, 2019a	hormone responsive BCP	52 (2)	48.8 (8.6)	HIIT UC	Serum microRNA level	12 weeks	RCT
Lee, 2019a	BCP undergoing chemotherapy	30 (0)	46.9 (9.8)	HIIT UC	VO_2max_, Peak power output	9 weeks	RCT (pilot)
Lee, 2019b	BCP undergoing chemotherapy	30 (0)	46.9 (9.8)	HIIT UC	Endothelial function, Vascular wall thickness	8 weeks	RCT
Alizadeh, 2019b	hormone responsive BCP	52 (2)	48.8 (8.6)	HIIT UC	Blood‐borne biomarkers	12 weeks	RCT
Lee, 2020	BCP undergoing chemotherapy	30 (0)	46.9 (9.8)	HIIT UC	MMP and TIMP	8 weeks	RCT (pilot)
Toohey, 2020	BCS	17 (0)	62.9 (7.8)	HIIT MICT UC	Cardiovascular fitness, Heart rate variability, Salivary biomarkers	12 weeks	RCT
Wiggenraad, 2020	BCP undergoing chemptherapy	206 (29)	53.3 (10.0)	AT‐HIIT RT‐HIIT UC	Symptom clusters were composed using the Memorial Symptom Assessment Scale (MSAS)	16 weeks	RCT
Bell, 2021	BCS after a rehabilitation program	20 (0)	50 (4.5)	HIIT MICT	Cardiovascular fitness, Body composition	12 weeks	RCT
Ochi, 2021	BCS	50 (6)	48.5 (5.5)	HIIT UC	VO_2peak_, Cardiorespiratory fitness, Muscle strength	12 weeks	RCT
Lee, 2021	BCP undergoing anthracycline‐based chemotherapy	30 (0)	46.9 (9.8)	HIIT UC	Patient‐reported outcomes, Physical function	8 weeks	RCT (pilot)
Hiensch, 2021	BCP undergoing adjuvant chemptherapy	86 (0)	53.0 (9.3)	AT‐HIIT RT‐HIIT UC	Blood‐borne outcomes, Fatigue, Muscle strength, Cardiorespiratory fitness, Body composition	16 weeks	RCT (pilot)
Moghadam, 2021	BCS	45 (5)	57 (1)	HIIT MICT UC	Blood‐borne outcomes, Body composition and physical fitness, Dietary intake	12 weeks	RCT
Samhan, 2021	overweight and obese BCS	63 (3)	49.3 (8.3)	HIIT UC	VO_2peak_, Body composition	8 weeks	RCT

Abbreviations: AT‐HIIT, moderate‐intensity aerobic and high‐intensity interval training; BCP, breast cancer patient; BCS, breast cancer survivor; HIIT, high‐intensity interval training; MICT, moderate‐intensity continuous training; QoL, quality of life; RT‐HIIT, resistance and high‐intensity interval training.

Among the 10 studies included in our review, a total number of *n* = 532 participants (mean age 54.05 ± 9.36 years) were included. Specifically, *n* = 296 (52.27 ± 9.49 years), *n* = 40 (56.66 ± 7.49 years), and *n* = 196 (51.57 ± 9.19 years) received HIIT, MICT, and UC, respectively. The duration of HIIT intervention ranged from 6 to 16 weeks, with an average of 10.4 (3.07) weeks. And the average number of weekly HIIT sessions was 2.7 (0.46).

All participants were women with Stage I–III breast cancer without metastases, recurrence, or secondary cancers at the time of enrollment. Except for two studies that recruited patients undergoing chemotherapy^36^, ^44^ and one study that did not specify limitations,^33^ the other eligible participants were mostly breast cancer survivors who had completed primary treatment, excluding hormone therapy. All studies excluded participants with cardiovascular diseases, hypertension, and any other contraindications to the exercise training program.

Intervention compliance was primarily evaluated by the percentage of attendance at arranged training sessions, which was generally high for both HIIT and MICT groups, except for the study by Mijwel et al., in which the attendance rates of the RT‐HIIT and AT‐HIIT groups were 68% and 63%, respectively. Training adherence for HIIT ranged from 78.7% to 100%, and for MICT, the rate ranged from 79.4% to 98.6%. The overall dropout rate across all studies was 8.89% (53/596), with HIIT at 7.59% (24/316), MICT at 4.76% (2/42), and UC at 12.22% (27/221). The dropout rates of individual studies ranged from a minimum of 0% to a maximum of 14.17% (34/240).^35^, ^36^

### 3.2 Risk of bias assessment

**FIGURE 2:**
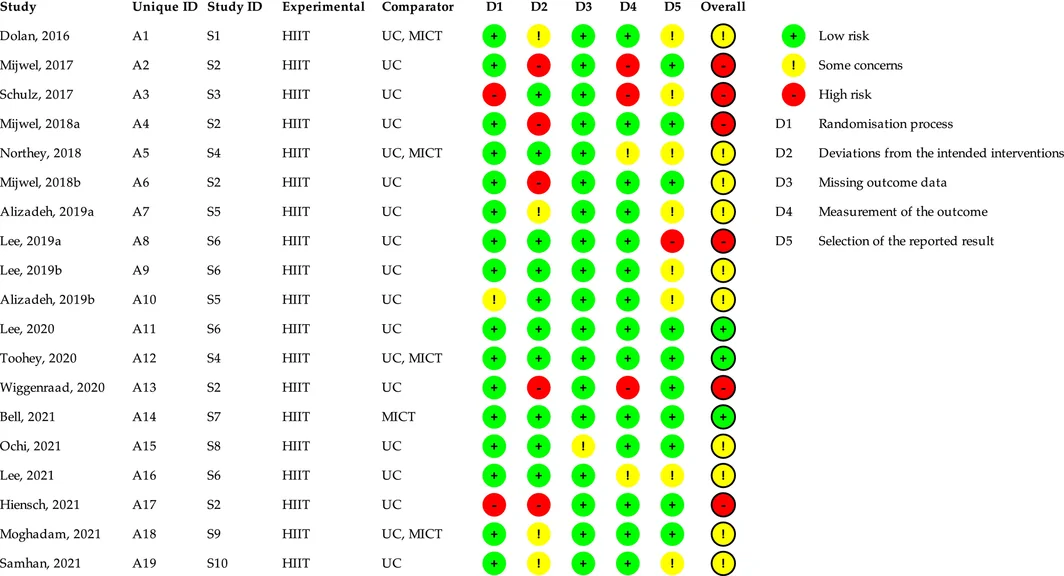
Bias evaluation of all included studies by the RoB 2 (risk of bias) evaluation tool. UC, usual care; MICT, moderate‐intensity continuous training; HIIT, high‐intensity interval training.

The results of the risk of bias assessment for all included studies are presented in Figure 2. The majority of studies reported a valid randomization procedure, with computer‐generated allocation sequences. However, two studies were assessed as having a high risk of randomization bias due to the lack of appropriate randomization procedures.^31^, ^33^ It was nearly impossible to blind participants due to the nature of the interventions, but those deviations from the intended interventions were usually balanced between groups and therefore were not likely to have affected the outcomes. However, in the Optitrain trial, there was a significant difference in the decline numbers between the intervention and control groups after randomization, indicating a high risk of bias for domain two.^30^, ^31^, ^34^, ^36^, ^41^ Three articles focused on patient‐reported outcomes were assessed as having a high risk of bias in the measurement of the outcomes.^30^, ^33^, ^41^ This was because these three articles used self‐reported questionnaires to exhibit one of their outcomes, and the interventions were impossible to keep the participants blinded. The predicted influence of the unavoidable bias might overestimate the advantages of HIIT slightly. One study was also found to have a high risk of reporting bias due to a discrepancy between the statistical analysis plans.^38^

### 3.3 Intervention effects and pooled analysis

The pooled estimates of health‐related outcomes between HIIT and MICT or UC are summarized in Table 2. Specific results that were reported only once will be described in a systematic review format to provide a comprehensive overview of the findings.

**TABLE 2:** The pooled estimates of health‐related outcomes between HIIT and MICT or UC.

Category	Outcomes	Comparative	Study number	Case number	SMD (95% CI)	*p*‐value*	Heterogeneity	
							*I* ^2^, %	*p*
Physical fitness	VO_2peak_ (L/min)	UC	2	176	0.79 (0.13, 1.45)	0.020*	72.4	0.057
	VO_2peak_ (mL/kg/min)	UC	4	143	0.59 (0.01, 1.16)	0.047*	61.1	0.052
	VO_2peak_ (mL/kg/min)	MICT	3	57	−0.05 (−0.58, 0.47)	0.839	0	0.473
	Handgrip strength (surgery site)	UC	2	176	0.18 (−0.30, 0.66)	0.462	51.7	0.150
	Strength 1RM	UC	2	66	0.76 (−0.28, 1.81)	0.153	72.2	0.058
	6MWT	UC	2	74	0.24 (−0.82, 1.29)	0.663	79.7	0.027
	STS test	UC	2	74	0.23 (−0.22, 0.69)	0.316	0	0.399
Body composition	Body mass	UC	3	219	−0.51 (−1.13, 0.11)	0.108	76.4	0.015
	Body mass	MICT	2	46	−0.08 (−0.66, 0.50)	0.786	0	0.561
	Fat mass	UC	2	87	−0.27 (−0.98, 0.44)	0.449	59.2	0.117
	Fat mass	MICT	2	46	−0.29 (−0.87, 0.29)	0.332	0	0.833
	Lean mass	UC	2	87	0.32 (−0.20, 0.83)	0.227	26.6	0.243
	Lean mass	MICT	2	46	0.13 (−0.45, 0.71)	0.659	0	0.729
Blood‐borne biomarkers	IL‐6	UC	3	131	−0.72 (−1.81, 0.38)	0.198	88.2	0.000
	IL‐10	UC	3	131	−0.54 (−1.74, 0.66)	0.376	90.2	0.000

Abbreviations: 6MWT, six‐minute walk test; CI, confidence interval; HIIT, high‐intensity interval training; IL, interleukin; MICT, moderate‐intensity continuous training; RM, repetition maximum; SMD, standard mean difference; STS, sit‐to‐stand; UC, usual care; VO_2peak_, peak oxygen uptake.

* *p* < 0.05 indicates statistical significance.

#### 3.3.1 Physical fitness

**FIGURE 3:**
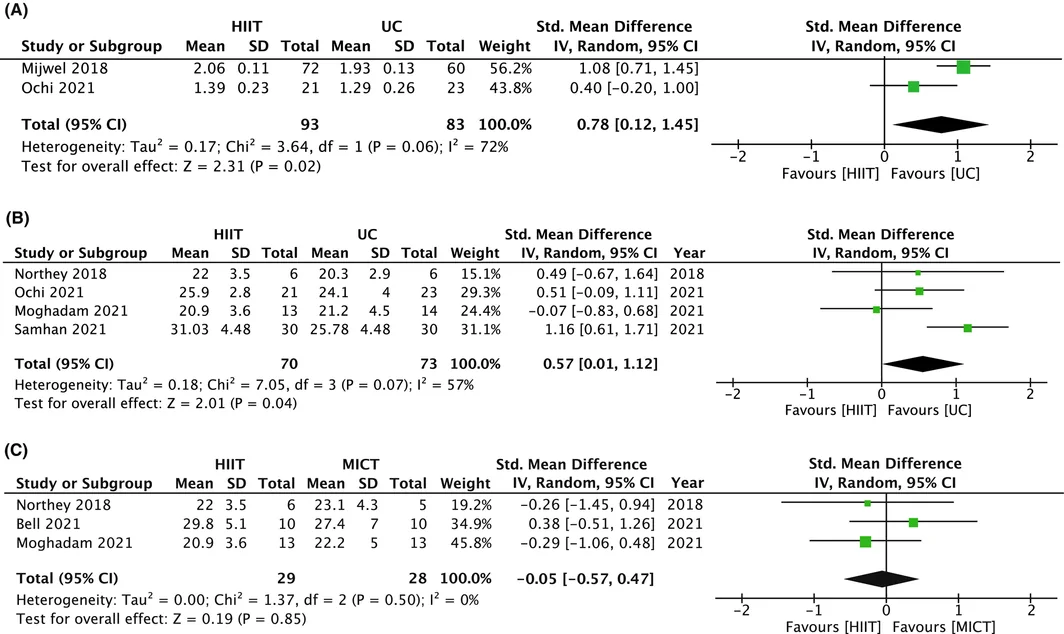
(A) Effect of HIIT and UC intervention on peak oxygen uptake (VO_2peak_) (L/min). (B) Effect of HIIT and UC intervention on VO_2peak_ (mL/kg/min). (C) Effect of HIIT and MICT intervention on VO_2peak_ (mL/kg/min). UC, usual care; MICT, moderate‐intensity continuous training; HIIT, high‐intensity interval training.

Several studies have investigated the impact of HIIT on VO_2peak_. Two studies compared post‐intervention VO_2peak_ (L/min) in different randomized groups, and the pooled result was 0.78 (95% CI 0.12, 1.45; *p* = 0.02, Figure 3A), suggesting that HIIT improves VO_2peak_ (L/min) compared to UC.^36^, ^43^ Four articles focused on the effect of HIIT on post‐intervention VO_2peak_ (mL/kg/min) between the HIIT group and the UC group.^35^, ^43^, ^45^, ^46^ The pooled result was 0.57 (95% CI 0.01, 1.12; *p* = 0.04, Figure 3B). However, the meta‐analysis indicated that there was no significant increase in VO_2peak_ (mL/kg/min) between the HIIT group and the MICT group (SMD = −0.05, 95% CI −0.57, 0.47; *p* = 0.47, Figure 3C).^35^, ^42^, ^45^ In addition, some articles also used the difference between post‐ and pre‐intervention VO_2peak_ as their outcome. For instance, Dolan et al. found that HIIT could significantly increase VO_2peak_ difference (mL/kg/min) [AIT: 11.48 (10.5); CMT: 12.95 (10.4); UC: −5.97 (7.2), *p* < 0.001].^32^

In terms of physical function, the six‐minute walk test (6MWT) was a commonly used endpoint for evaluating functional performance. Two articles compared the results of the 6MWT between the HIIT and control groups.^43^, ^44^ However, the pooled results showed no significant improvement in the 6MWT after HIIT (SMD = 0.24, 95% CI −0.82, 1.30; *p* = 0.663). For the sit‐to‐stand (STS) test, the estimated pooled SMD was 0.23 (95% CI −0.22, 0.69; *p* = 0.316). Additionally, Lee et al. utilized the timed up and go (TUG) and Margaria–Kalamen stair test, which indicated that no significant difference between groups was observed in the TUG test (−0.45, 95% CI −1.65, 0.74; *p* = 0.52).^44^ The results for the stair test showed that the time taken in the HIIT group was lower than that in the UC group (−1.08, 95% CI −2.49, 0.33; *p* = 0.013).

Regarding muscle strength, two studies compared handgrip strength between the HIIT and UC groups for the surgery side.^36^, ^43^ The estimated pooled SMD was 0.18 (95% CI −0.30, 0.66; *p* = 0.462). Two other studies compared strength measured by one‐repetition maximum (1RM) between the HIIT and control groups.^32^, ^43^ Significant heterogeneity (*I* ^2^ = 72.2%) was reported, and the pooled result overlapped zero (SMD = 0.76, 95% CI −0.28, 1.81; *p* = 0.153). Another study assessed both lower body strength (LBS) and upper body strength (UBS), and found a significant increase in both HIIT and MICT groups.^45^ And ANOVA showed that the increase in LBS was more pronounced in the HIIT group than MICT or UC group. Additionally, Schulz et al. conducted a combined HIIT/strength training, and the strength performance (mean change of cumulative load 25.9% ± 11.2%) significantly increased in the HIIT group compared to UC.^33^

As for cardiovascular fitness, Northey et al. examined several indices like middle artery mean blood flow velocity (MCAvmean) and mean arterial pressure (MAP).^35^ They found that HIIT had a large effect on these measures. Another study used brachial artery flow‐mediated dilation (baFMD) to assess vascular endothelial function and found a significant increase in baFMD in the HIIT group compared to both the UC group and baseline measurements.^29^ However, carotid intima media thickness (cIMT) showed no significant difference after the intervention (within‐group mean change: −0.003 mm, 95% CI −0.004, 0.009; between‐group *p* = 0.23). Additionally, peak workload, minute ventilation, and peak heart rate were also used to assess cardiovascular fitness, but no significant differences were observed either between or within groups.^42^

Moreover, Mijwel et al. conducted a biopsy analysis to compare the skeletal muscle fiber characteristics between cancer patients undergoing HIIT and those in the UC group.^34^ They found that HIIT helped to preserve the function and features of muscle fibers, as demonstrated by higher citrate synthase activity (HIIT vs. UC: *p* = 0.005), improved activity of oxphos complexes (HIIT vs. UC: complex I: *p* = 0.003; complex II: *p* = 0.007; complex IV: *p* = 0.004), and greater cross‐sectional area (HIIT vs. control: *p* = 0.02).

#### 3.3.2 Body composition

Three studies investigating the effect of HIIT on body mass indicated no significant decrease compared to the UC group (SMD = −0.51, 95% CI −1.13, 0.11; *p* = 0.108).^30^, ^45^, ^46^ Similarly, the HIIT group showed no significant improvement in body mass control compared to the MICT group (SMD = −0.08, 95% CI −0.66, 0.50; *p* = 0.786).^42^, ^45^ However, Dolan et al. found that both HIIT and MICT could help in body mass control (AIT: −0.67 (1.9); CMT: −0.41 (2.08); UC: 1.44 (1.62), *p* = 0.031).^32^

Two articles comparing post‐intervention fat mass between the HIIT and the UC group showed an estimated SMD of −0.27 (95% CI −0.98, 0.44; *p* = 0.449).^45^, ^46^ Similarly, only two articles investigated the different effects of HIIT and MICT on fat mass, and the results also overlapped zero (SMD = −0.29, 95% CI −0.87, 0.29; *p* = 0.332).^42^, ^45^

In respect of lean mass, two articles compared the effect of HIIT and the UC group, and the pooled result showed no significant difference between the two groups (SMD = 0.32, 95% CI −0.20, 0.83; *p* = 0.227).^45^, ^46^ Two articles comparing HIIT and MICT also showed no significant difference (SMD = 0.13, 95% CI −0.45, 0.71; *p* = 0.66).^42^, ^45^

Waist and hip circumference were also used to assess body composition, and Dolan et al. found that these indices decreased significantly in the HIIT group compared to the UC group.^32^ The appendicular lean muscle index did not show any significant differences either between groups or within groups.

#### 3.3.3 Blood‐borne biomarkers

The impact of HIIT on blood‐borne biomarkers has been investigated by several studies. Three articles reported on the effect of HIIT on IL‐6 levels compared to UC, with a SMD estimate of −0.72 (95% CI −1.81, 0.38; *p* = 0.198).^31^, ^39^, ^45^ Additionally, these three articles indicated that HIIT had an effect on IL‐10 levels compared to UC, with a pooled SMD estimate of −0.54 (95% CI −1.74, 0.66; *p* = 0.376).

However, Dolan et al. explored other blood‐borne biomarkers and observed no significant differences in insulin, glucose, or hs‐CRP levels between groups.^32^ Lee et al. demonstrated a significant within‐group decrease in matrix metalloproteinases‐9 levels following HIIT intervention [104.3 (51.9)–65.2 (69.1); −37.4%; *p* = 0.01; *d* = 0.20].^40^

Hiensch et al. compared a large cluster of blood‐borne biomarkers between and within groups.^31^ The results showed that FasL and CXCL9 levels increased after HIIT intervention (FasL: Mean = 0.31, 95% CI 0.13, 0.48; *p* < 0.05; CXCL9: Mean = 0.66, 95% CI 0.32, 1.00; *p* < 0.05), and the levels of CD40‐L, EGF, CCL17, and CASP‐8 attenuated significantly after UC, while the levels of TRAIL, DCN, ISOSLG, CSF‐1, FasL, and CXCL9 increased in the UC group. However, there was no significant difference between HIIT intervention and UC in terms of all inflammatory markers.

Moghadam et al. reported significant reductions in TNF‐α and leptin levels in both the HIIT and MICT groups, while adiponectin levels were significantly increased in both groups.^45^ Further ANOVA analysis revealed that the reduction in TNF‐α and leptin levels was more prominent in the HIIT group.

Alizadeh et al. monitored serum microRNA (miR) levels in patients undergoing hormone therapy.^37^ They found that the expression levels of cancer‐related miRs (oncomiRs) decreased in the HIIT group (miR‐21: 25 [0.5]; *p* = 0.018; miR‐155: 1.3 [0.4]; *p* = 0.005; miR‐27a: 1.5 [0.02]; *p* = 0.037; miR‐10b: 1.95 [0.6]; *p* = 0.031), while the level of miR‐221 was not significantly influenced by HIIT (2.8 [0.4]; *p* = 0.137). The study also found that the levels of several tumor suppressor miRs (TsmiRs) were upregulated after HIIT (miR‐206: 3.01 [0.5]; *p* = 0.008; miR‐145: 6.9 [0.2]; *p* = 0.001; miR‐143: 6.1 [0.5]; *p* = 0.023; let‐7a: 2.6 [0.25]; *p* = 0.036), except for miR‐9 (4.3 [0.5]; *p* = 0.566).

#### 3.3.4 Patient‐reported outcomes

Several studies have used patient‐reported outcome measures to evaluate the effects of HIIT on cancer patients. Mijwel et al. compared cancer‐related fatigue (CRF) between HIIT and UC group using the Piper Fatigue Scale (PFS).^30^ Self‐reported results showed that CRF increased significantly in the UC group compared with the HIIT group. Similarly, Ochi et al. used various assessment tools, including the Global Physical Activity Questionnaire and Cancer Fatigue Scale to evaluate patient‐reported outcomes.^43^ They found that HIIT could benefit breast cancer patients in terms of fatigue (ES = 0.50, *p* = 0.09).

HRQoL was measured by the European Organization for Research and Treatment of Cancer Quality of Life Questionnaire (EORTC‐QLQ‐C30), and Mijwel et al. found that emotional function was higher in the HIIT group, while negative physical function and pain scores were relatively lower.^30^ In line with the emotional benefits, Schulz et al. measured anxiety and depression with the Hospital Anxiety and Depression Scale (German Version) and found that HIIT could decrease both anxiety and depression levels.^33^ Additionally, Northey et al. measured cognitive performance with the CogState battery and found that the HIIT group had moderate to large effect sizes for executive function and working memory.^35^

Symptoms and symptom burden were measured by the Memorial Symptom Assessment Scale (MSAS) after a 16‐week intervention of AT‐HIIT or RT‐HIIT in the Optitrain trial.^30^, ^41^ Mijwel et al. found that the score in the UC group increased significantly, while the score in the HIIT group stayed unchanged. Moreover, at the end of the 12‐month follow‐up, Wiggenraad et al. once again applied MSAS to assess burdensome symptoms in the emotional, treatment‐related toxicity, and physical dimensions. They reported that the score of “feeling sad” in the emotional dimension was lower in the HIIT group compared with UC after the intervention (−0.13, 95% CI −0.23, −0.03; *p* < 0.05), and the score of “feeling irritable” in the emotional dimension was also lower in the HIIT group (−0.20, 95% CI −0.34, −0.07; *p* < 0.05). No other significant differences were found.

Furthermore, Lee et al. used the Functional Assessment of Cancer Therapy‐Breast Cancer (FACT‐B), 15‐item Five‐Facet Mindfulness Questionnaire (FFMQ‐15), and Multidimensional Fatigue Inventory with 20 questions (MDFI‐20) to assess patient‐reported outcomes.^44^ The results showed that only the score on FACT‐B physical, functional, and total well‐being decreased significantly in the UC group, while no significant difference was found between groups after the intervention.

## 4 DISCUSSION

The objective of this systematic review and meta‐analysis was to investigate the potential beneficial effects of HIIT on health‐related outcomes in breast cancer patients compared to MICT and UC groups. Our pooled analysis of 10 RCTs indicated that HIIT led to a significant improvement in cardiorespiratory fitness (VO_2peak_) compared to the UC group, but no significant differences were observed between the MICT group and HIIT. Moreover, no significant differences were found in other physical fitness indices, body composition, and blood‐borne biomarkers between the MICT and UC groups. Furthermore, based on our systematic review, it was observed that HIIT may also enhance muscle strength, reduce CRF, and alleviate emotional symptoms, although further empirical evidence is still required. In the subsequent sections, we will interpret and summarize the findings, as well as discuss future directions for investigating the effects of HIIT in breast cancer patients.

### 4.1 Outcomes interpretations

For physical fitness, in terms of cardiorespiratory function, our pooled results revealed that HIIT led to significantly greater improvements in VO_2peak_ compared to UC, while the difference between HIIT and MICT was negligible. These findings align with previous systematic reviews and meta‐analyses that have demonstrated the positive effects of HIIT on cardiorespiratory fitness in cancer patients and survivors across various cancer types, including lung cancer and mixed types.^21^, ^22^, ^26^, ^47^, ^48^ Improved cardiorespiratory fitness, as measured by VO_2peak_, is clinically important as it has been widely accepted as a strong predictor of decreased total cancer mortality risk and better health outcomes.^49^, ^50^ Our findings suggest that both HIIT and MICT can help prevent the decline in cardiorespiratory fitness during breast cancer treatment or aftercare.

We found that the performance of physical function tests did not differ between the HIIT and UC groups, as the two relevant studies generated opposite results.^43^, ^44^ However, with respect to muscle strength, all three trials that investigated the indicators of lower limb strength elicited a significant increase in the HIIT group.^32^, ^43^, ^45^ These results are consistent with a review suggesting that HIIT could improve lower limb strength in healthy older adults.^51^ Previous studies have shown that lower body strength in breast cancer patients during or after chemotherapy treatment is lower than the norms in the general population and is correlated with an increased risk of falls and fractures.^52^, ^53^ Therefore, we suggest that HIIT is beneficial for preventing sarcopenia and reducing the risk of falls in the breast cancer population.^53^, ^54^ Moreover, Schulz et al. observed significant improvements in strength capacity among breast cancer patients who underwent a combination of HIIT and standard strength training.^33^ Although distinguishing the isolated effects of HIIT from those of strength training presents a challenge, this study serves as an inspiration for further investigation, which should compare the effects of HIIT plus resistance training versus strength training alone to determine whether the combination of HIIT and strength training yields synergistic effects in improving muscle strength.

Meanwhile, multiple indices were utilized to evaluate the effects of HIIT on cardiovascular fitness. In one study, an 8‐week HIIT intervention resulted in significant improvements in baFMD and the maintenance of cIMT in breast cancer patients receiving anthracycline‐based chemotherapy, compared to significant reductions in baFMD and increases in cIMT in patients not receiving HIIT.^29^ However, in two other studies involving breast cancer survivors, there were no significant differences in cardiovascular‐related outcomes between the HIIT and UC groups.^35^, ^42^ It is well‐known that anthracyclines negatively alter vascular endothelial function and wall thickness, leading to cardiovascular diseases.^55^, ^56^, ^57^ Thus, the reason for this discrepancy may be that patients undergoing chemotherapy were more negatively influenced by anthracycline, and HIIT partially offset the drug side‐effects. Collectively, we suggest that initiating HIIT exercise as early as possible after diagnosis, particularly during chemotherapy, can yield enhanced benefits for breast cancer patients compared to commencing exercise after treatment. This hypothesis aligns with the primary recommendation for physical activity as outlined in the ACS/ASCO 2016 Breast Cancer Survivorship Care Guideline.^12^

Only three studies have investigated the effects of HIIT on body composition,^30^, ^45^, ^46^ and our pooled analysis showed no significant differences between HIIT and control groups in terms of body mass, fat mass, or lean mass. However, all three studies reported significant differences in body mass, indicating that HIIT prevented body weight increase compared to UC group. The inconsistency in findings may be explained by the differences in statistical methodology and huge heterogeneity. In our meta‐analysis, we compared post‐intervention results between groups, while two studies only compared within‐group differences across the HIIT and control groups. Furthermore, the risk of bias assessment revealed that the baseline characteristics of the three studies may not be completely comparable since the *p* values were not reported, which may have affected the pooled results. In contrast to our findings, a recent review on the effects of HIIT in cancer patients found significant reductions in fat mass after HIIT intervention.^26^ However, this review included patients with mixed types of cancer, and the results may not be generalizable to breast cancer patients.

Numerous studies have examined changes in blood‐borne biomarkers resulting from HIIT interventions^31^, ^32^, ^37^, ^39^, ^40^, ^45^; however, the heterogeneity of indices across studies has hindered further pooled analysis. Three studies monitored the levels of both IL‐6 and IL‐10, and the estimated pooled results indicated no significant difference between HIIT and UC groups.^31^, ^39^, ^45^ While clusters of indices such as inflammatory markers and microRNAs have been assessed,^31^, ^37^ a large proportion of between‐group analyses failed to identify any significant differences. Only one study observed significantly decreased levels of TNF‐α and leptin and increased adiponectin concentration in the HIIT group,^45^ which has been associated with a lower risk of obesity and breast cancer mortality.^58^, ^59^ The importance of HIIT for breast cancer patients may stem in part from its anti‐inflammatory mechanisms, achieved through the down‐regulation of pro‐inflammatory factors such as IL‐6, and the up‐regulation of anti‐inflammatory factors like IL‐10 and TNF‐α. This significance arises from the direct association between inflammation and tumor growth, as well as the potential protective effects against chemotoxicity and treatment‐related cardiovascular toxicity.

Six studies investigated patient‐reported outcomes, including HRQoL and CRF.^30^, ^33^, ^35^, ^41^, ^43^, ^44^ However, the use of heterogeneous evaluation tools prevented quantitative synthesis between studies. While two studies found that HIIT could benefit breast cancer patients in terms of fatigue,^30^, ^43^ another study reported no significant between‐group difference was spotted.^44^ HRQoL was also a notable outcome assessed by five studies,^30^, ^33^, ^35^, ^41^, ^44^ and the results favored HIIT in terms of improved emotional well‐being, including alleviated symptoms of anxiety, depression, sadness, irritation, poor working memory, and increased executive function compared to the UC group. The physical and psychological decline caused by cancer or cancer treatment‐related factors is strongly linked to impaired HRQoL.^60^, ^61^ Our findings are consistent with two concurrent reviews, which suggest that HIIT may have positive effects on CRF and HRQoL for cancer patients.^26^, ^48^ The positive effects of HIIT on patient‐reported outcomes were evident across various assessment scales, as indicated by the findings of our systematic review. Encouraging patients to engage in HIIT during treatment is of importance, as it can help prevent a progressive decline in their overall health and well‐being.

### 4.2 Study limitations

This study presents the first meta‐analysis results examining the effects of HIIT in breast cancer patients, covering a broad range of health‐related outcomes to provide a comprehensive review. However, there are several limitations that should be taken into account when interpreting the findings. Firstly, the number of studies included was relatively low, and some articles were based on a same clinical trial. Secondly, due to the scarcity of RCTs focusing on HIIT in breast cancer patients, the quality of randomized control trials was not strictly controlled, which could have introduced potential bias. Thirdly, the stage, subtype, and treatment of breast cancer patients were heterogeneous, which may impact the generalizability of the findings. Fourthly, while we analyzed numerous health‐related outcomes, some specific indices were only available in a limited number of articles, and a large proportion of these indices could not be combined, leading to the application of qualitative analysis and preventing further sensitivity or subgroup analysis.

### 4.3 Future orientations

While this study supports the benefits of HIIT in breast cancer patients in terms of VO_2peak_, further RCTs are still necessary to substantiate this crucial significance in larger cohorts. Given the small sample size and inconsistent results observed for other health outcomes, additional evidence is warranted to determine the effects of HIIT on physical function, cardiovascular fitness, body compositions, blood‐borne biomarkers and patient‐reported outcomes in breast cancer patients.

In particularly, greater attention should be directed towards body composition. Women with breast cancer commonly gain about 5 kg body weight during chemotherapy, and few return to their pre‐diagnosis weight.^62^ Since weight gain is associated with comorbidities and recurrence,^63^ maintaining pre‐diagnosis weight is of major importance. Previous reviews have demonstrated that HIIT significantly reduced fat mass and body weight in overweight or obese adults.^64^, ^65^ However, few studies have focused on examining body composition changes during and after HIIT intervention specifically in breast cancer patients. Therefore, further research on these outcomes is necessary.

Moreover, we observed a lack of uniformity in the indices utilized to assess physical function and cardiovascular fitness, as well as significant heterogeneity in blood‐borne biomarkers and HRQoL assessment tools. Consequently, there is a need to develop a standardized study protocol or guideline to effectively address this issue. Additionally, as previously discussed, the hypothesized anti‐inflammatory mechanisms of HIIT may potentially offer protective effects against treatment‐related cardiovascular toxicity. To gain a deeper understanding of the molecular mechanisms involved, it is recommended to conduct further investigations on blood‐borne biomarkers and their correlations with cardiovascular fitness indices.

In terms of participant selection, future studies should consider dividing breast cancer patients into two groups: those undergoing chemotherapy and survivors. Given that adjuvant therapies (chemotherapy, radiotherapy, and hormone therapy) can negatively impact health outcomes and have long‐lasting effects, it is important to differentiate the effect size of HIIT between these two conditions. Furthermore, exploring the effectiveness of HIIT across different cancer types and treatment time points should be considered as a future direction in the field of supportive medicine in cancer care.

## 5 CONCLUSIONS

Our study suggests that HIIT is an effective alternative strategy to MICT, leading to significant improvements in VO_2peak_ as compared to UC. HIIT may also improve muscle strength and alleviate CRF and emotional symptoms. However, the evidence is limited regarding the effects of HIIT on other outcomes. We recommend that HIIT could be considered as a feasible exercise intervention for breast cancer patients during and after treatment. Further studies with larger cohorts are needed to determine the clinical significance of HIIT‐induced changes in body composition, cardiovascular fitness, and blood‐borne biomarkers in women with breast cancer.

## AUTHOR CONTRIBUTIONS

**Xudong Chen:** Conceptualization (lead); formal analysis (equal); investigation (lead); methodology (equal); project administration (lead); resources (equal); supervision (equal); validation (equal); writing – original draft (lead); writing – review and editing (equal). **Xuyuan Shi:** Conceptualization (supporting); formal analysis (equal); investigation (equal); project administration (equal); resources (equal); validation (equal); writing – original draft (equal); writing – review and editing (equal). **Zhiruo Yu:** Formal analysis (supporting); investigation (supporting); validation (supporting); writing – original draft (equal); writing – review and editing (supporting). **Xuelei Ma:** Conceptualization (supporting); investigation (supporting); project administration (supporting); validation (equal); writing – review and editing (supporting).

## FUNDING INFORMATION

This research received no external funding.

## CONFLICT OF INTEREST STATEMENT

The authors declare no conflict of interest. We are not using any copyrighted information, patient photographs, identifiers, or other protected health information in this paper. No text, text boxes, figures, or tables in this article have been previously published or owned by another party.

## Supporting information


Table SA1.

Table SA2.

## Data Availability

The original contributions presented in the study are included in the article, and the data will be available at the request of the author.

## References

[1] Sung H , Ferlay J , Siegel RL , et al. Global cancer statistics 2020: GLOBOCAN estimates of incidence and mortality worldwide for 36 cancers in 185 countries. CA Cancer J Clin. 2021;71(3):209‐249. doi:10.3322/caac.21660 33538338

[2] Allemani C , Matsuda T , Di Carlo V , et al. Global surveillance of trends in cancer survival 2000‐14 (CONCORD‐3): analysis of individual records for 37 513 025 patients diagnosed with one of 18 cancers from 322 population‐based registries in 71 countries. Lancet. 2018;391(10125):1023‐1075. doi:10.1016/S0140-6736(17)33326-3 29395269 PMC5879496

[3] Friese CR , Harrison JM , Janz NK , et al. Treatment‐associated toxicities reported by patients with early‐stage invasive breast cancer. Cancer. 2017;123(11):1925‐1934. doi:10.1002/cncr.30547 28117882 PMC5444953

[4] Franzoi MA , Agostinetto E , Perachino M , et al. Evidence‐based approaches for the management of side‐effects of adjuvant endocrine therapy in patients with breast cancer. Lancet Oncol. 2021;22(7):e303‐e313. doi:10.1016/S1470-2045(20)30666-5 33891888

[5] Javan Biparva A , Raoofi S , Rafiei S , et al. Global quality of life in breast cancer: systematic review and meta‐analysis. BMJ Support Palliat Care. 2022;bmjspcare‐2022‐003642. doi:10.1136/bmjspcare-2022-003642 PMC10850719 35710706

[6] Shapiro CL . Cancer survivorship. N Engl J Med. 2018;379(25):2438‐2450. doi:10.1056/NEJMra1712502 30575480

[7] Ammitzboll G , Sogaard K , Karlsen RV , et al. Physical activity and survival in breast cancer. Eur J Cancer. 2016;66:67‐74. doi:10.1016/j.ejca.2016.07.010 27529756

[8] McTiernan A , Friedenreich CM , Katzmarzyk PT , et al. Physical activity in cancer prevention and survival: a systematic review. Med Sci Sports Exerc. 2019;51(6):1252‐1261. doi:10.1249/MSS.0000000000001937 31095082 PMC6527123

[9] Furmaniak AC , Menig M , Markes MH . Exercise for women receiving adjuvant therapy for breast cancer. Cochrane Database Syst Rev. 2016;9(9):CD005001. doi:10.1002/14651858.CD005001.pub3 27650122 PMC6457768

[10] Lahart IM , Metsios GS , Nevill AM , Carmichael AR . Physical activity for women with breast cancer after adjuvant therapy. Cochrane Database Syst Rev. 2018;1(1):CD011292. doi:10.1002/14651858.CD011292.pub2 29376559 PMC6491330

[11] Rezende LFM , Sa TH , Markozannes G , et al. Physical activity and cancer: an umbrella review of the literature including 22 major anatomical sites and 770 000 cancer cases. Br J Sports Med. 2018;52(13):826‐833. doi:10.1136/bjsports-2017-098391 29146752

[12] Runowicz CD , Leach CR , Henry NL , et al. American Cancer Society/American Society of Clinical Oncology breast cancer survivorship care guideline. CA Cancer J Clin. 2016;66(1):43‐73. doi:10.3322/caac.21319 26641959

[13] Buffart LM , Galvao DA , Brug J , Chinapaw MJ , Newton RU . Evidence‐based physical activity guidelines for cancer survivors: current guidelines, knowledge gaps and future research directions. Cancer Treat Rev. 2014;40(2):327‐340. doi:10.1016/j.ctrv.2013.06.007 23871124

[14] Cormie P , Zopf EM , Zhang X , Schmitz KH . The impact of exercise on cancer mortality, recurrence, and treatment‐related adverse effects. Epidemiol Rev. 2017;39(1):71‐92. doi:10.1093/epirev/mxx007 28453622

[15] Scott JM , Li N , Liu Q , et al. Association of Exercise with Mortality in adult survivors of childhood cancer. JAMA Oncol. 2018;4(10):1352‐1358. doi:10.1001/jamaoncol.2018.2254 29862412 PMC6181767

[16] Kampshoff CS , van Dongen JM , van Mechelen W , et al. Long‐term effectiveness and cost‐effectiveness of high versus low‐to‐moderate intensity resistance and endurance exercise interventions among cancer survivors. J Cancer Surviv. 2018;12(3):417‐429. doi:10.1007/s11764-018-0681-0 29497963 PMC5956032

[17] Buchheit M , Laursen PB . High‐intensity interval training, solutions to the programming puzzle: part I: cardiopulmonary emphasis. Sports Med. 2013;43(5):313‐338. doi:10.1007/s40279-013-0029-x 23539308

[18] Taylor JL , Holland DJ , Spathis JG , et al. Guidelines for the delivery and monitoring of high intensity interval training in clinical populations. Prog Cardiovasc Dis. 2019;62(2):140‐146. doi:10.1016/j.pcad.2019.01.004 30685470

[19] Blackwell JEM , Doleman B , Herrod PJJ , et al. Short‐term (<8 wk) high‐intensity interval training in diseased cohorts. Med Sci Sports Exerc. 2018;50(9):1740‐1749. doi:10.1249/MSS.0000000000001634 29683925 PMC6133203

[20] Weston KS , Wisloff U , Coombes JS . High‐intensity interval training in patients with lifestyle‐induced cardiometabolic disease: a systematic review and meta‐analysis. Br J Sports Med. 2014;48(16):1227‐1234. doi:10.1136/bjsports-2013-092576 24144531

[21] Wallen MP , Hennessy D , Brown S , et al. High‐intensity interval training improves cardiorespiratory fitness in cancer patients and survivors: a meta‐analysis. Eur J Cancer Care (Engl). 2020;29(4):e13267. doi:10.1111/ecc.13267 32469144

[22] Lavin‐Perez AM , Collado‐Mateo D , Mayo X , et al. High‐intensity exercise to improve cardiorespiratory fitness in cancer patients and survivors: a systematic review and meta‐analysis. Scand J Med Sci Sports. 2021;31(2):265‐294. doi:10.1111/sms.13861 33098219

[23] Herranz‐Gomez A , Cuenca‐Martinez F , Suso‐Marti L , et al. Effectiveness of HIIT in patients with cancer or cancer survivors: an umbrella and mapping review with meta‐meta‐analysis. Scand J Med Sci Sports. 2022;32(11):1522‐1549. doi:10.1111/sms.14223 35925829 PMC9804206

[24] Page MJ , McKenzie JE , Bossuyt PM , et al. The PRISMA 2020 statement: an updated guideline for reporting systematic reviews. BMJ. 2021;372:n71. doi:10.1136/bmj.n71 33782057 PMC8005924

[25] Borg GA . Psychophysical bases of perceived exertion. Med Sci Sports Exerc. 1982;14(5):377‐381. 7154893

[26] Mugele H , Freitag N , Wilhelmi J , et al. High‐intensity interval training in the therapy and aftercare of cancer patients: a systematic review with meta‐analysis. J Cancer Surviv. 2019;13(2):205‐223. doi:10.1007/s11764-019-00743-3 30806875

[27] Sterne JAC , Savovic J , Page MJ , et al. RoB 2: a revised tool for assessing risk of bias in randomised trials. BMJ. 2019;366:l4898. doi:10.1136/bmj.l4898 31462531

[28] Toohey K , Pumpa K , McKune A , et al. The impact of high‐intensity interval training exercise on breast cancer survivors: a pilot study to explore fitness, cardiac regulation and biomarkers of the stress systems. BMC Cancer. 2020;20(1):787. doi:10.1186/s12885-020-07295-1 32819304 PMC7441660

[29] Lee K , Kang I , Mack WJ , et al. Effects of high‐intensity interval training on vascular endothelial function and vascular wall thickness in breast cancer patients receiving anthracycline‐based chemotherapy: a randomized pilot study. Breast Cancer Res Treat. 2019;177(2):477‐485. doi:10.1007/s10549-019-05332-7 31236810 PMC6661195

[30] Mijwel S , Backman M , Bolam KA , et al. Adding high‐intensity interval training to conventional training modalities: optimizing health‐related outcomes during chemotherapy for breast cancer: the OptiTrain randomized controlled trial. Breast Cancer Res Treat. 2018;168(1):79‐93. doi:10.1007/s10549-017-4571-3 29139007 PMC5847033

[31] Hiensch AE , Mijwel S , Bargiela D , Wengstrom Y , May AM , Rundqvist H . Inflammation mediates exercise effects on fatigue in patients with breast cancer. Med Sci Sports Exerc. 2021;53(3):496‐504. doi:10.1249/MSS.0000000000002490 32910094 PMC7886356

[32] Dolan LB , Campbell K , Gelmon K , Neil‐Sztramko S , Holmes D , McKenzie DC . Interval versus continuous aerobic exercise training in breast cancer survivors—a pilot RCT. Support Care Cancer. 2016;24(1):119‐127. doi:10.1007/s00520-015-2749-y 25957010

[33] Schulz SVW , Laszlo R , Otto S , et al. Feasibility and effects of a combined adjuvant high‐intensity interval/strength training in breast cancer patients: a single‐center pilot study. Disabil Rehabil. 2018;40(13):1501‐1508. doi:10.1080/09638288.2017.1300688 28325109

[34] Mijwel S , Cardinale DA , Norrbom J , et al. Exercise training during chemotherapy preserves skeletal muscle fiber area, capillarization, and mitochondrial content in patients with breast cancer. FASEB J. 2018;32(10):5495‐5505. doi:10.1096/fj.201700968R 29750574

[35] Northey JM , Pumpa KL , Quinlan C , et al. Cognition in breast cancer survivors: a pilot study of interval and continuous exercise. J Sci Med Sport. 2019;22(5):580‐585. doi:10.1016/j.jsams.2018.11.026 30554923

[36] Mijwel S , Backman M , Bolam KA , et al. Highly favorable physiological responses to concurrent resistance and high‐intensity interval training during chemotherapy: the OptiTrain breast cancer trial. Breast Cancer Res Treat. 2018;169(1):93‐103. doi:10.1007/s10549-018-4663-8 29349712 PMC5882634

[37] Alizadeh S , Isanejad A , Sadighi S , Khalighfard S , Alizadeh AM . Effect of a high‐intensity interval training on serum microRNA levels in women with breast cancer undergoing hormone therapy. A single‐blind randomized trial. Ann Phys Rehabil Med. 2019;62(5):329‐335. doi:10.1016/j.rehab.2019.07.001 31400480

[38] Lee K , Kang I , Mack WJ , et al. Feasibility of high intensity interval training in patients with breast cancer undergoing anthracycline chemotherapy: a randomized pilot trial. BMC Cancer. 2019;19(1):653. doi:10.1186/s12885-019-5887-7 31269914 PMC6610838

[39] Alizadeh AM , Isanejad A , Sadighi S , Mardani M , Kalaghchi B , Hassan ZM . High‐intensity interval training can modulate the systemic inflammation and HSP70 in the breast cancer: a randomized control trial. J Cancer Res Clin Oncol. 2019;145(10):2583‐2593. doi:10.1007/s00432-019-02996-y 31401675 PMC11810413

[40] Lee K , Kang I , Mack WJ , et al. Effect of high intensity interval training on matrix metalloproteinases in women with breast cancer receiving anthracycline‐based chemotherapy. Sci Rep. 2020;10(1):5839. doi:10.1038/s41598-020-61927-x 32246106 PMC7125197

[41] Wiggenraad F , Bolam KA , Mijwel S , van der Wall E , Wengstrom Y , Altena R . Long‐term favorable effects of physical exercise on burdensome symptoms in the OptiTrain breast cancer randomized controlled trial. Integr Cancer Ther. 2020;19:1534735420905003. doi:10.1177/1534735420905003 32090630 PMC7040931

[42] Bell RA , Baldi JC , Jones LM . Additional cardiovascular fitness when progressing from moderate‐ to high‐intensity exercise training in previously trained breast cancer survivors. Support Care Cancer. 2021;29(11):6645‐6650. doi:10.1007/s00520-021-06259-w 33956212

[43] Ochi E , Tsuji K , Narisawa T , et al. Cardiorespiratory fitness in breast cancer survivors: a randomised controlled trial of home‐based smartphone supported high intensity interval training. BMJ Support Palliat Care. 2022;12(1):33‐37. doi:10.1136/bmjspcare-2021-003141 PMC8862092 34389552

[44] Lee K , Norris MK , Wang E , Dieli‐Conwright CM . Effect of high‐intensity interval training on patient‐reported outcomes and physical function in women with breast cancer receiving anthracycline‐based chemotherapy. Support Care Cancer. 2021;29(11):6863‐6870. doi:10.1007/s00520-021-06294-7 34018031

[45] Hooshmand Moghadam B , Golestani F , Bagheri R , et al. The effects of high‐intensity interval training vs. moderate‐intensity continuous training on inflammatory markers, body composition, and physical fitness in overweight/obese survivors of breast cancer: a randomized controlled clinical trial. Cancers. 2021;13(17):4386. doi:10.3390/cancers13174386 34503198 PMC8430701

[46] Samhan AF , Ahmed AS , Mahmoud WS , Abdelhalim NM . Effects of high‐intensity interval training on cardiorespiratory fitness, body composition, and quality of life in overweight and obese survivors of breast cancer. Rehabil Oncol. 2021;39(4):168‐174. doi:10.1097/01.Reo.0000000000000270

[47] Heredia‐Ciuro A , Fernandez‐Sanchez M , Martin‐Nunez J , et al. High‐intensity interval training effects in cardiorespiratory fitness of lung cancer survivors: a systematic review and meta‐analysis. Support Care Cancer. 2022;30(4):3017‐3027. doi:10.1007/s00520-021-06647-2 34714414

[48] Palma S , Hasenoehrl T , Jordakieva G , Ramazanova D , Crevenna R . High‐intensity interval training in the prehabilitation of cancer patients‐a systematic review and meta‐analysis. Support Care Cancer. 2021;29(4):1781‐1794. doi:10.1007/s00520-020-05834-x 33106975 PMC7892520

[49] Schmid D , Leitzmann MF . Cardiorespiratory fitness as predictor of cancer mortality: a systematic review and meta‐analysis. Ann Oncol. 2015;26(2):272‐278. doi:10.1093/annonc/mdu250 25009011

[50] Myers J , McAuley P , Lavie CJ , Despres JP , Arena R , Kokkinos P . Physical activity and cardiorespiratory fitness as major markers of cardiovascular risk: their independent and interwoven importance to health status. Prog Cardiovasc Dis. 2015;57(4):306‐314. doi:10.1016/j.pcad.2014.09.011 25269064

[51] Elboim‐Gabyzon M , Buxbaum R , Klein R . The effects of high‐intensity interval training (HIIT) on fall risk factors in healthy older adults: a systematic review. Int J Environ Res Public Health. 2021;18(22):11809. doi:10.3390/ijerph182211809 34831565 PMC8618957

[52] Neil‐Sztramko SE , Kirkham AA , Hung SH , Niksirat N , Nishikawa K , Campbell KL . Aerobic capacity and upper limb strength are reduced in women diagnosed with breast cancer: a systematic review. J Physiother. 2014;60(4):189‐200. doi:10.1016/j.jphys.2014.09.005 25443649

[53] Winters‐Stone KM , Nail L , Bennett JA , Schwartz A . Bone health and falls: fracture risk in breast cancer survivors with chemotherapy‐induced amenorrhea. Oncol Nurs Forum. 2009;36(3):315‐325. doi:10.1188/09.ONF.315-325 19596649

[54] Liu QQ , Xie WQ , Luo YX , et al. High intensity interval training: a potential method for treating sarcopenia. Clin Interv Aging. 2022;17:857‐872. doi:10.2147/CIA.S366245 35656091 PMC9152764

[55] Kalabova H , Melichar B , Ungermann L , et al. Intima‐media thickness, myocardial perfusion and laboratory risk factors of atherosclerosis in patients with breast cancer treated with anthracycline‐based chemotherapy. Med Oncol. 2011;28(4):1281‐1287. doi:10.1007/s12032-010-9593-1 20567943

[56] Okur A , Karadeniz C , Ozhan Oktar S , Pinarli FG , Aral A , Oguz A . Assessment of brachial artery reactivity, carotid intima‐media thickness, and adhesion molecules in pediatric solid tumor patients treated with anthracyclines. Pediatr Hematol Oncol. 2016;33(3):178‐185. doi:10.3109/08880018.2016.1146375 26984313

[57] Xu JZ , Wu SY , Yan YQ , et al. Left atrial diameter, flow‐mediated dilation of brachial artery and target organ damage in Chinese patients with hypertension. J Hum Hypertens. 2012;26(1):41‐47. doi:10.1038/jhh.2010.129 21289644

[58] Atoum MF , Alzoughool F , Al‐Hourani H . Linkage between obesity leptin and breast cancer. Breast Cancer (Auckl). 2020;14:1178223419898458. doi:10.1177/1178223419898458 31975779 PMC6956603

[59] Wulaningsih W , Holmberg L , Ng T , Rohrmann S , Van Hemelrijck M . Serum leptin, C‐reactive protein, and cancer mortality in the NHANES III. Cancer Med. 2016;5(1):120‐128. doi:10.1002/cam4.570 26632325 PMC4708908

[60] Muhandiramge J , Orchard SG , Warner ET , van Londen GJ , Zalcberg JR . Functional decline in the cancer patient: a review. Cancers (Basel). 2022;14(6):1368. doi:10.3390/cancers14061368 35326520 PMC8946657

[61] Mosconi P , Colozza M , De Laurentiis M , De Placido S , Maltoni M . Survival, quality of life and breast cancer. Ann Oncol. 2001;12(Suppl 3):S15‐S19. doi:10.1093/annonc/12.suppl_3.s15 11804378

[62] Jiralerspong S , Goodwin PJ . Obesity and breast cancer prognosis: evidence, challenges, and opportunities. J Clin Oncol. 2016;34(35):4203‐4216. doi:10.1200/JCO.2016.68.4480 27903149

[63] Demark‐Wahnefried W , Schmitz KH , Alfano CM , et al. Weight management and physical activity throughout the cancer care continuum. CA Cancer J Clin. 2018;68(1):64‐89. doi:10.3322/caac.21441 29165798 PMC5766382

[64] Maillard F , Pereira B , Boisseau N . Effect of high‐intensity interval training on total, abdominal and visceral fat mass: a meta‐analysis. Sports Med. 2018;48(2):269‐288. doi:10.1007/s40279-017-0807-y 29127602

[65] Bellicha A , van Baak MA , Battista F , et al. Effect of exercise training on weight loss, body composition changes, and weight maintenance in adults with overweight or obesity: an overview of 12 systematic reviews and 149 studies. Obes Rev. 2021;22(Suppl 4):e13256. doi:10.1111/obr.13256 33955140 PMC8365736

