# Supplementary material for: High‐intensity interval training in breast cancer patients: A systematic review and meta‐analysis
Source: Cancer Med. 2023 Aug 17;12(17):17692–705. doi: 10.1002/cam4.6387 (PMC10524023; doi:10.1002/cam4.6387)
Supplement: Supplementary file 1 — Table SA1. [file CAM4-12-17692-s002.docx]

**Supplementary Table A1.** Literature search results

**Embase <1974 to 2022 November 07>**

| Search number | Query | Results |
| --- | --- | --- |
| 1 | mammary.ti,ab. | 88607 |
| 2 | breast.ti,ab. | 683179 |
| 3 | exp breast/ | 124612 |
| 4 | exp breast disease/ | 648937 |
| 5 | 1 or 2 or 3 or 4 | 876076 |
| 6 | exp neoplasm/ | 5235111 |
| 7 | tumor*.ti,ab. | 2235247 |
| 8 | tumour*.ti,ab. | 412520 |
| 9 | neoplas*.ti,ab. | 387348 |
| 10 | carcinoma*.ti,ab. | 971250 |
| 11 | malignan*.ti,ab. | 925454 |
| 12 | sarcoma*.ti,ab. | 127610 |
| 13 | lymphoma*.ti,ab. | 290335 |
| 14 | DCIS.ti,ab. | 10751 |
| 15 | LCIS.ti,ab. | 1210 |
| 16 | 6 or 7 or 8 or 9 or 10 or 11 or 12 or 13 or 14 or 15 | 6008979 |
| 17 | 5 and 16 | 713643 |
| 18 | exp breast cancer/ | 536703 |
| 19 | 17 or 18 | 713643 |
| 20 | high intensity.ti,ab. | 41884 |
| 21 | exp aerobic exercise/ | 20911 |
| 22 | aerobic.ti,ab. | 116260 |
| 23 | sprint.ti,ab. | 9573 |
| 24 | 20 or 21 or 22 or 23 | 168990 |
| 25 | exp interval training/ | 4340 |
| 26 | interval.ti,ab. | 855396 |
| 27 | intermittent.ti,ab. | 127292 |
| 28 | 26 or 27 | 977084 |
| 29 | exp training/ | 103088 |
| 30 | training.ti,ab. | 672427 |
| 31 | exp exercise/ | 404754 |
| 32 | exercise*.ti,ab. | 447412 |
| 33 | 29 or 30 or 31 or 32 | 1178059 |
| 34 | 28 and 33 | 50110 |
| 35 | 25 or 34 | 50936 |
| 36 | 24 and 35 | 9854 |
| 37 | 19 and 36 | 165 |

**Pubmed <1974 to 2022 November 07>**

| Search number | Query | Results |
| --- | --- | --- |
| 1 | "mammary"[Title/Abstract] | 77,158 |
| 2 | "breast"[Title/Abstract] | 500,145 |
| 3 | "breast"[MeSH Terms] | 51,826 |
| 4 | "breast diseases"[MeSH Terms] | 350,467 |
| 5 | #1 OR #2 OR #3 OR #4 | 602,974 |
| 6 | "neoplasms"[MeSH Terms] | 3,753,306 |
| 7 | "cancer*"[Title/Abstract] | 2,153,982 |
| 8 | "tumor*"[Title/Abstract] | 1,689,792 |
| 9 | "tumour*"[Title/Abstract] | 299,847 |
| 10 | "neoplas*"[Title/Abstract] | 439,283 |
| 11 | "carcinoma*"[Title/Abstract] | 753,036 |
| 12 | "malignan*"[Title/Abstract] | 662,001 |
| 13 | "sarcoma"[Title/Abstract] | 92,641 |
| 14 | "lymphoma"[Title/Abstract] | 181,747 |
| 15 | "DCIS"[Title/Abstract] | 5,777 |
| 16 | "LCIS"[Title/Abstract] | 607 |
| 17 | #5 OR #6 OR #7 OR #8 OR #9 OR #10 OR #11 OR #12 OR #13 OR #14 OR #15 OR #16 | 5,040,973 |
| 18 | #5 AND #17 | 602,974 |
| 19 | "High-Intensity Interval Training"[MeSH Terms] | 1,947 |
| 20 | "high intensity"[Title/Abstract] | 34,501 |
| 21 | "aerobic"[Title/Abstract] | 98,620 |
| 22 | "sprint"[Title/Abstract] | 8,800 |
| 23 | #20 OR #21 OR #22 | 137,258 |
| 24 | "interval"[Title/Abstract] | 685,274 |
| 25 | "intermittent"[Title/Abstract] | 92,743 |
| 26 | #24 OR #25 | 773,776 |
| 27 | "training*"[Title/Abstract] | 519,432 |
| 28 | "exercise*"[Title/Abstract] | 345,869 |
| 29 | #27 OR #28 | 798,540 |
| 30 | #23 AND #26 AND #29 | 7,590 |
| 31 | #18 AND #30 | 108 |

**Web of Science <1974 to 2022 November 07>**

| # | Query | Results |
| --- | --- | --- |
| 1 | (TS=(mammary)) OR TS=(breast) | 800438 |
| 2 | ((((((((TS=(neoplasm*)) OR TS=(tumor*)) OR TS=(tumour*)) OR TS=(carcinoma*)) OR TS=(malignan*)) OR TS= (sarcoma*)) OR TS= (lymphoma*)) OR TS=(DCIS)) OR TS=(LCIS) | 3041510 |
| 3 | TS=(breast cancer) | 597590 |
| 4 | (#1 and #2) or #3 | 660697 |
| 5 | ((TS=(high-intensity)) OR TS=(aerobic)) OR TS=(sprint) | 209884 |
| 6 | (TS=(interval)) OR TS=(intermittent) | 1164877 |
| 7 | (TS=(training*)) OR TS=(exercise*) | 905731 |
| 8 | TS=(high-intensity interval training) | 4417 |
| 9 | TS=(HIIT) | 1765 |
| 10 | (#5 and #6 and #7) or #8 or #9 | 11203 |
| 11 | #10 AND #4 | 180 |
